# Monocular Camera/IMU/GNSS Integration for Ground Vehicle Navigation in Challenging GNSS Environments

**DOI:** 10.3390/s120303162

**Published:** 2012-03-07

**Authors:** Tianxing Chu, Ningyan Guo, Staffan Backén, Dennis Akos

**Affiliations:** 1 School of Earth and Space Sciences, Peking University, No. 5 Yiheyuan Road, Haidian District, Beijing 100871, China; 2 School of Instrumentation Science and Optoelectronics Engineering, Beihang University, Xueyuan Road No.37, Haidian District, Beijing 100191, China; E-Mail: ningyan.guo@gmail.com; 3 Department of Aerospace Engineering Sciences, University of Colorado at Boulder, Boulder, CO 80309, USA; E-Mails: staffan.backen@ltu.se (S.B.); dma@colorado.edu (D.A.)

**Keywords:** sensor integration, extended Kalman filter, GNSS, strapdown mechanization, computer vision, tightly coupled integration

## Abstract

Low-cost MEMS-based IMUs, video cameras and portable GNSS devices are commercially available for automotive applications and some manufacturers have already integrated such facilities into their vehicle systems. GNSS provides positioning, navigation and timing solutions to users worldwide. However, signal attenuation, reflections or blockages may give rise to positioning difficulties. As opposed to GNSS, a generic IMU, which is independent of electromagnetic wave reception, can calculate a high-bandwidth navigation solution, however the output from a self-contained IMU accumulates errors over time. In addition, video cameras also possess great potential as alternate sensors in the navigation community, particularly in challenging GNSS environments and are becoming more common as options in vehicles. Aiming at taking advantage of these existing onboard technologies for ground vehicle navigation in challenging environments, this paper develops an integrated camera/IMU/GNSS system based on the extended Kalman filter (EKF). Our proposed integration architecture is examined using a live dataset collected in an operational traffic environment. The experimental results demonstrate that the proposed integrated system provides accurate estimations and potentially outperforms the tightly coupled GNSS/IMU integration in challenging environments with sparse GNSS observations.

## 1. Introduction

GNSS systems have been the prominent technology to fulfill the demanding requirements for road navigation. However, GNSS signals are subject to attenuation, reflections, or even blockages as a result of driving in difficult signal reception areas, such as urban canyons, enclosed parking garages and underground concrete tunnels. RF interference (RFI), whether deliberate or not, can misguide the user towards an erroneous position solution [1]. The use of augmentation technologies is capable of mitigating these disadvantages of standalone GNSS solutions and providing improved availability and accuracy. By coupling the measurements from both GNSS and IMU sensors, integration techniques are commercially employed to calculate a high-bandwidth navigation solution. This effectively enables the calibration of the IMU sensors and the control of the error growth of inertial navigation, and also enables higher data output compared to a standalone GNSS solution. A number of researchers have presented detailed realization methods and performance analyses of the loosely/tightly coupled GNSS/IMU implementations [2–4].

Apart from the traditional navigation technologies, the progress in computer vision has motivated a variety of location-based applications, such as simultaneous localization and mapping (SLAM) [5], and provided potential capabilities for the navigation community. Visual sensors usually incorporate other sensors, such as GNSS, IMU and laser scanners, to achieve a system level integration. Soloviev [6] provided an integration of GPS carrier phase and vision-based measurements for navigation in GPS challenged environments. The GPS carrier phase measurements were used to resolve the scale ambiguity of the unknown change in position vector. The algorithm was validated via simulations as well as live experiments.

In more common cases, a vision sensor is incorporated with an IMU sensor for motion estimation with high frequency. Corke [7] presented a tutorial overview on inertial and visual sensors and gave the essential principles of integrating them to obtain robust estimates of sensor motion and 3D reconstruction. The integration strategy is categorized into the loose and tight coupling. A detailed performance comparison of the loosely/tightly coupled camera/IMU integration was given by Chu [8]. The tightly coupled integration might yield higher accuracy, but it is prone to divergence under certain conditions and *vice versa*.

By utilizing strapdown inertial sensors and a fisheye lens, Schlaile [9] demonstrated a navigation system for an indoor vertical-take-off-and-landing (VTOL) micro aerial vehicle (MAV) based on the Kalman filter. The inverse depth method was applied to determine the movement of the camera. The scale factor ambiguity was recovered by simulating the laser ranging as additional source of information and measuring the flight height over ground. Apart from using a monocular camera, Soloviev [10] proposed a multi-aperture approach integrated with IMU measurements. By observing the feature points over a wide range, the vision-related navigation uncertainty could be significantly reduced. The unit sphere on which the feature points were mapped was presented and used to model the sensor fusion algorithm.

As opposed to utilizing a forward-looking camera, Hide [11] used a camera with a ground-facing orientation to accomplish the camera/IMU integration for pedestrian navigation. Given the estimated height to the ground, the camera velocity was retrieved from the translation information. The residual between IMU and camera velocities then served as the Kalman filter’s measurements. With a similar IMU/camera mounting method, Huang [12] provided a tightly coupled integration by using the residual between the predicted and actual feature location as the filter’s input, which was expressed in the image coordinate frame.

Nowadays, low-cost MEMS-based IMUs, video cameras and portable GNSS devices are commercially available for automotive applications and some manufacturers have already integrated such facilities into their vehicle systems. This research aims at robust ground vehicle navigation based on camera/IMU/GNSS integration against challenging GNSS environments where the blocked sky can significantly obstruct the view of low-elevation-angle satellites and hinder a user’s navigation performance.

Three key contributions are addressed herein: first, the proposed camera/IMU/GNSS integration architecture complements each of the sensors and potentially overcomes the disadvantage of traditional GNSS/IMU integration. For example, the number of line-of-sight (LOS) GNSS pseudoranges remains between two and three for a while such as wandering through the narrow streets in a city’s downtown area. Although the tightly coupled GNSS/IMU integration allows the filter to obtain the user’s position solution with sparse measurements, the overall solution is prone to divergence over time due to the lack of redundant constraints. On the other hand, with the aid of more than two LOS pseudorange observations from high-elevation-angle satellites, the baseline magnitude of position change within an allocated time interval can be extracted. This GNSS-derived baseline magnitude in turn enables a monocular camera to resolve its scale factor ambiguity if the data rates of GNSS and camera sensors are synchronized.

Second, although significant effort has been spent on developing visual odometry algorithms, the efficiency and performance regarding image frame rate has yet to be evaluated. The navigation information derived from the computer vision algorithms is, ideally, independent of frame rate, provided two adjacent image frames overlap each other. However, in reality the actual situation does not provide this independence due to the vehicle dynamics and sufficiency of the accurate image feature correspondences. In an effort to balance the tradeoff between practical performance and computational cost, we deliberately reduce the image frame rate and examine the positioning accuracy of our integrated scheme.

Third, the integrated system is validated through a live dataset which was collected in an operational traffic environment with available centimeter-accuracy truth reference. Although there are previous usable datasets for SLAM-based analysis, few of them were collected in the actual environment with available high-accuracy truth reference [13,14]. The camera images we used were captured in an operational traffic environment with vehicles passing by and overtaking the host vehicle during the test period. The high-accuracy truth reference was generated by leveraging three RTK GPS receivers on the vehicle roof top, which facilitates a fair and reliable comparison between algorithms under assessment. The only simulation in this paper is the GNSS pseudorange measurements.

The remainder of the paper proceeds as follows: we first introduce the coordinate frames utilized in this paper. The inertial navigation and its error model are then summarized. Thirdly, the principle of computer vision-based motion estimation is presented. GNSS simulation and differential processing are discussed afterwards. Based on the above sensor description, the proposed EKF model and integrated architecture are established, followed by the live test validation. The paper concludes with the results as well as planned future work.

## 2. Coordinate Frames

**Figure 1.**
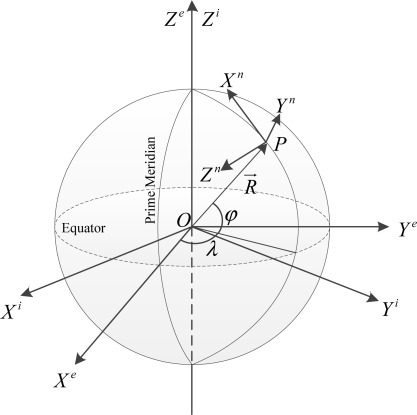
Inertial frame, earth frame and navigation frame representation on the Earth ellipsoid.

Figure 1 illustrates three major coordinate frames on the Earth ellipsoid. The inertial coordinate frame (*i* frame) *O-X^i^Y^i^Z^i^* is the fundamental reference frame for inertial navigation and an IMU enclosure usually measures the specific force as well as the angular rate relative to the *i* frame. Its origin is on the Earth’s center of mass *O* and it is stationary relative to the fixed stars. We usually express the ultimate position solution, latitude *φ*, longitude *λ* and height *h*, in the Earth frame (*e* frame) *O-X^e^Y^e^Z^e^*. Its origin is the Earth’s center of mass *O*, with *Z^e^* axis forming along polar axis and *X^e^* axis lying on the intersection of the equatorial plane and prime meridian plane. The *Y^e^* axis is also on the equatorial plane and satisfies the right-hand rule. The origin and Z-axes of the *i* and *e* frames are coincident, respectively. In this paper, the *e* frame-based position solutions are based on the WGS-84 ellipsoid. To describe the velocity as well as the orientation of a ground vehicle, the right-handed navigation frame (*n* frame) *P*-*X^n^Y^n^Z^n^* is utilized and locally placed at the user’s position *P*. The *Z^n^* axis is collinear with the local normal line of the reference ellipsoid pointing downwards while *X^n^* and *Y^n^* axes indicate the local north and east direction, respectively.

**Figure 2.**
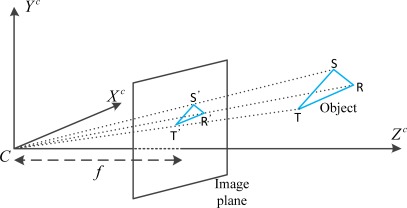
Representation of the camera frame with respect to the pinhole projection model.

The specific force $f_{\mathrm{ib}}^{b}$ and angular rate $\omega_{\mathrm{ib}}^{b}$ from an IMU are usually measured relative to the *i* frame and expressed in the body frame (*b* frame). The *b* frame has its origin at the center of the IMU enclosure. If the IMU is mounted parallel to the vehicle frame, we allocate the *X^b^* axis pointing forward, in view of the vehicle, and *Z^b^* axis aligning with local gravity direction. The *Y^b^* axis satisfies the right-hand rule and indicates the right-side direction of the vehicle. Based on the pinhole projection model in our research, the image feature points are expressed in the camera frame (*c* frame) which is extended from the 2D image plane. The *c* frame representation is shown in Figure 2. The *c* frame’s origin is at the camera center *C*, and *X^c^* as well as *Y^c^* axes point towards left and upward directions of the image plane, correspondingly. The *Z^c^* axis lies along the principal axis and is orthogonal to the image plane. *f* denotes the focal length of the camera.

In this paper, the vehicle dynamics model is established in terms of the *b* frame instead of other frames. According to Hol [15], the *b* frame based motion estimate has less modeling complexity and is prone to eliminating the influence of angular and position installation errors.

## 3. Inertial Navigation and IMU Error Model

A six-degree-of-freedom (6-DOF) strapdown IMU provides the user high-bandwidth position, velocity and orientation estimation with complete independence of the reception of electromagnetic waves as following differential equations [16]:

(1) $${\dot{C}}_{b}^{n}=C_{b}^{n}\cdot (\omega_{\mathrm{ib}}^{b}\times )-[(\omega_{\mathrm{ie}}^{n}\times )+(\omega_{\mathrm{en}}^{n}\times )]\cdot C_{b}^{n}$$

(2) $${\dot{V}}^{n}=C_{b}^{n}f_{\mathrm{ib}}^{b}-[2(\omega_{\mathrm{ie}}^{n}\times )+(\omega_{\mathrm{en}}^{n}\times )]\cdot V^{n}+g^{n}(\varphi ,h)$$

(3) $${\dot{P}}^{e}=\mathrm{diag}(\frac{1}{R_{M}+h}\;\frac{1}{(R_{N}+h)\;\text{cos}\varphi }\;-1)\cdot V^{n}$$

In Equation (1), $C_{b}^{n}$ is the direction cosine matrix representing the rotation between the *b* and *n* frames. The variables $\omega_{\mathrm{ie}}^{n}$ and $\omega_{\mathrm{en}}^{n}$ are the Earth’s rotation rate and body transport rate, respectively. The symbol × denotes the skew symmetric matrix of a specific angular rate term. The calculated $C_{b}^{n}$ is then used to estimate the acceleration in terms of the *n* frame by multiplication with measured specific force $f_{\mathrm{ib}}^{b}$. As described in Equation (2), Coriolis force, body transport rate and local gravity terms are compensated in the estimation. Although the local gravity changes with latitude *φ* and height *h*, the insensitivity can be ignored for a ground vehicle. In Equation (3), the position update is expressed in the *e* frame and therefore needs *R_M_* and *R_N_*, radii of curvatures in the meridian and prime vertical, to be estimated.

Although a strapdown IMU propagates the high-frequency inertial navigation mathematically derived from Equations (1) to (3), the MEMS accelerometer and gyroscope sensors inevitably drift and give rise to quadratic and cubic accumulated mechanization errors. Researchers have developed error propagation models in various approaches for practical applications such as [17–19]. To describe a ground vehicle in low dynamics with well-calibrated a priori information, the Ψ angle model [20] is introduced in this paper on account of simplicity:

(4) $${\delta \dot{P}}^{n}=-(\omega_{\mathrm{en}}^{n}\times ){\delta P}^{n}+{\delta V}^{n}$$

(5) $$\begin{array}{lll} {\delta \dot{V}}^{n} & = & \mathrm{diag}[-\frac{g}{R_{e}}\;-\frac{g}{R_{e}}\;\frac{2g}{R_{e}}]{\delta P}^{n}-[({2\omega }_{\mathrm{ie}}^{n}+\omega_{\mathrm{en}}^{n})\times ]{\delta V}^{n}\\ & & +[(C_{b}^{n}f_{\mathrm{ib}}^{b})\times ]{\delta \Psi }^{n}+C_{b}^{n}{\delta f}^{b} \end{array}$$

(6) $$\delta {\dot{\Psi }}^{n}=-(\omega_{\mathrm{in}}^{n}\times )\delta \Psi^{n}-C_{b}^{n}\delta \omega^{b}$$

In the above equations, *R_e_* is the Earth radius; *δP^n^*, *δV^n^*, *δ*Ψ*^n^*, *δf^b^* and *δω^b^* are the perturbation terms of position, velocity, orientation and sensor biases, respectively.

As noted earlier, this paper aims to efficiently integrate data from camera, IMU and GNSS sensors to achieve high accuracy ground vehicle navigation in challenging GNSS environments. Therefore, a model for reasonably calibrating the IMU sensor biases needs to be established considering the practical situation. A commonly cited first-order Gauss-Markov model regards the IMU errors as 3 independent components: a random constant, a Gauss-Markov variable and a white noise term and therein the accelerometer/gyroscope error propagations are written as following equations [16,18]:

(7) $$\delta f=\delta f_{c}+\delta f_{m}+w_{f}\;\;\delta \omega ={\delta \omega }_{c}+{\delta \omega }_{m}+w_{\omega }$$

The subscripts of *c* and *m* indicate the random constant and first-order Gauss-Markov terms, and their derivatives yield:

(8) $$\delta {\dot{f}}_{c}=0\;\;\delta {\dot{\omega }}_{c}=0$$

(9) $$\delta {\dot{f}}_{m}=-\frac{\delta f_{m}}{T_{f}}+\mu_{f}\;\;\delta {\dot{\omega }}_{m}=-\frac{{\delta \omega }_{m}}{T_{\omega }}+\mu_{\omega }$$

where the constants *T_f_* and *T_w_* are the correlation times; *w_f_*, *w_w_*, *μ_f_* and *μ_w_* are assumed uncorrelated and correlated white Gaussian noise (WGN) for inertial sensors. Equations (4) to (9) construct the fundamental linearized dynamic system of the EKF integrated system which will be further discussed in Section 6.

## 4. Motion Estimation in Computer Vision

The camera requires calibration prior to the computer vision routine for motion estimation. To effectively estimate the intrinsic camera parameters as well as distortion characteristics of the images, researchers have developed practical calibration methods such as [21–23] and there exist open source toolkits for calibration-related tasks. Normally, the camera calibration parameters do not change rapidly over time, moreover, the run-to-run calibration differences are even negligible within a limited period of time, provided the camera is not reconfigured. In addition, as opposed to an IMU, a temperature-independent CMOS/CCD sensor makes the camera resistant to ambient environmental change. Therefore, compared to the inertial sensors, the camera exhibits different calibration mechanism and characteristics coupled with a diverse model for long-term stability.

As mentioned previously, the perspective projection with a pinhole camera model is used. To estimate the motion in successive image frames, the computer vision module executes three main functions: (1) feature extraction and matching; (2) outlier removal and discrimination of the moving features; (3) motion estimation.

### 4.1. Feature Extraction and Matching

Feature extraction is the essential process for subsequent motion estimation. As an outdoor environment usually has a high-contrast texture in the image scene, image points with distinct structural information are selected as features. In computer vision community, the scale-invariant feature transform (SIFT) algorithm [24] is a well-accepted approach to describe and match the feature points which are usually invariant to distortion, scaling, orientation, affine transformation, and illumination changes. Although several faster algorithms, such as PCA [25] and SURF [26], facilitate reducing the computational load, the point location accuracy can be in turn degraded [27]. We, consequently, use the SIFT algorithm to locate the feature points and create the corresponding scale-invariant descriptor vectors by measuring the gradient magnitude and orientation in the pre-defined pixel neighborhood. To provide sufficient dimensionalities in the feature space for favorable matching result, a 128-dimensional descriptor:

(10) $$\vec{d}={\left(d_{1},d_{2},\ldots ,d_{128}\right)}^{T}$$

is used for each pre-selected feature point. In adjacent images, the similarity of two candidate feature points, *A* and *B*, is given by the Euclidean distance:

(11) $$l_{\mathrm{AB}}\;=\;||{\vec{d}}_{A}-{\vec{d}}_{B}||\;=\;\sqrt{\sum_{i=1}^{128}\;{\delta d}_{i}^{2}}$$

By setting the empirically examined accept-reject threshold, the feature matching process is evaluated using the ratio of the minimum distance to the next minimum [24]. Ideally, if an image scene contains a non-uniform texture, there should be a large amount of SIFT features which guarantee desirable redundancy for optimized motion estimation. However, there inevitably exist outliers due to the limited descriptor dimensionality and the imperfect matching determination criteria. Therefore, an outlier removal scheme is undertaken prior to the motion determination process. In order to remove the outlier correspondences which potentially give rise to undesirable estimation, the random sample consensus (RANSAC) algorithm [28] is taken into consideration in this paper.

### 4.2. Outlier Removal by RANSAC

Based on the primary principle of epipolar geometry, all corresponding image points satisfy the coplanarity equation:

(12) $${x^{\prime }}^{T}\text{Fx}=0$$

where F is a 3 × 3 fundamental matrix which geometrically describes the projection between two corresponding points in a pair of images representing the same 3D object; x and x′ are the coordinates of the points in image planes, respectively. It is supposed that RANSAC algorithm estimates the fundamental matrix F which optimally fits all the inliers. However, RANSAC may miss out a few outliers and estimate a biased F matrix due to the improper number of iteration operations and a relatively large number of outliers. Consequently, a multilayer RANSAC scheme [29] is proposed and implemented as follows:

1. arbitrarily selecting a subset samples from all pre-selected matching features;
2. reconstructing epipolar geometry constraint satisfying Equation (12) and computing the fundamental matrix F based on the selected samples;
3. using the same constraint and the calculated matrix F to determine the error from all features;
4. with a proper threshold, categorizing all feature correspondences as inliers or outliers by appraising the error statistics;
5. iterating the first three steps multiple times (refer to [28] for a favorable number of iterations);
6. finding the best-fit matrix F which yields the optimal error estimates and removing the outliers discriminated by this matrix F;
7. repeating the first six steps with a gradually shrunken inlier/outlier determination threshold.

**Figure 3.**
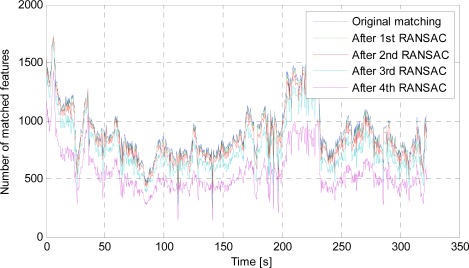
Number of matched features after implementing multilayer RANSAC.

To test the multilayer RANSAC scheme, we use a sequence of images (2.5 Hz rate) which will be elaborated in Section 7. Figure 3 shows the number of originally matched features as well as that after implementing multilayer RANSAC. It is clearly seen that after the fourth layer of RANSAC processing, there are still hundreds of matched features for every moment to maintain observable redundancy for motion estimation.

**Figure 4.**
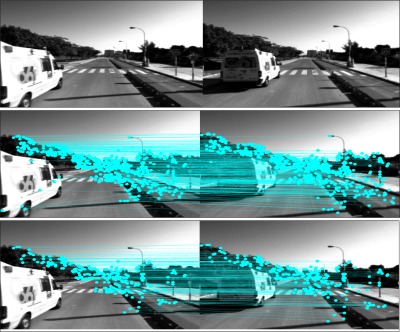
Feature matching and outlier removal between two image frames.

Figure 4 intuitively illustrates a demonstration of feature matching and outlier removal between two adjacent image frames. The upper two subfigures are the original images for analysis showing a van overtaking the host vehicle. The middle ones present the feature matching after SIFT processing, and each cyan line indicates a pair of feature correspondence. It can be noted that the false matching arises and the images thus require outlier removal algorithms in order to retain only the static inliers. The lower subplots present the refined matching results by applying multilayer RANSAC scheme. As it has shown, all the remainder feature correspondences are recognized as inliers and no feature locates on the moving van any more.

### 4.3. Motion Estimation

After the refinement of feature matching by implementing the multilayer RANSAC scheme, we also compute the most reasonable estimate of the matrix F. According to the primary principles of epipolar geometry and the properties of fundamental matrix [30], we have the following relationship between the motion of the camera and fundamental matrix:

(13) $$E=K^{T}\text{FK}=(\vec{l}\times )\cdot R$$

where *l⃗* × is the skew symmetric of camera translation; R is the rotation matrix; K contains the intrinsic calibration parameters and E is the essential matrix. By implementing the singular value decomposition (SVD), the camera rotation R and unit translation vector *l⃗* regarding two adjacent images can be finally retrieved and serve as the vision-based navigation parameters for integration purposes. It is worth noting that since the essential matrix E has only five degrees of freedom, the 3× 1 vector *l⃗* is resolved up to scale and is accordingly subject to an ambiguity issue in terms of the translation magnitude. If the absolute magnitude of the translation between two successive images is extracted by other approaches, which we will elaborate in Section 5, the scale ambiguity of *l⃗* can be finally resolved.

## 5. Determination of Translation Magnitude from GNSS Measurements

In an effort to resolve the scale ambiguity issue of a monocular camera, there are several available approaches, such as using the wheel tick and inertial sensors [6]. Before resorting to the inertial-aided approach, a decorrelation solution between sensor error and inertial mechanization is desired. An analogous situation applies to using a wheel tick sensor if its drift arises quickly. Conversely, as one of the most widely applied outdoor positioning technologies, GNSS does not yield accumulated ranging or positioning errors, and can provide the capability to resolve the scale ambiguity problem of a monocular camera by leveraging GNSS differential technique. If the acquired GNSS and image data are synchronized, the baseline of position change between two GNSS epochs coincides with the desired camera translation. The GNSS observation equation for pseudorange measurements is given by:

(14) $$P^{i}=\rho^{i}+c\cdot t_{r}-c\cdot T^{i}-d_{I}^{i}+d_{T}^{i}+\varepsilon^{i}\;\;\;\;\;i=1,...,k$$

where:

*P^i^* is the pseudorange measurement;
*ρ^i^* is the true geometric range between the designated GNSS satellite and the vehicle;
*t_r_* is the receiver clock error;
*T^i^* is the satellite clock error;
*c* is the speed of light;
$d_{I}^{i}$ and $d_{T}^{i}$ are ionospheric and tropospheric corrections, respectively, along the signal propagation path;
*ε^i^* denotes the unmodeled observation errors containing the thermal noise and so forth;

The superscript *i* indicates the satellite index and *k* represents the total tracked number of satellites.

Resorting to the concept of differential positioning technology, Equation (14) between two epochs yield differential expression as follows:

(15) $${\delta P}^{i}={\delta \rho }^{i}+c\cdot {\delta t}_{r}+{\delta \varepsilon }^{i}\;\;\;\;i=1,...,k$$

where the notation *δ* depicts the differential operation for each term. Under low dynamic conditions with short time intervals, the differential operation adequately eliminates the ionospheric and tropospheric effects. For the differential term of the satellite clock error, it can be either calculated using the decoded ephemeris or simply ignored as the satellite clock drift can be neglected in a short temporal scale.

According to van Graas [31] and Soloviev [6], the true range differential *δρ^i^* correlates the baseline magnitude of position change and the relationship satisfies:

(16) $$\begin{array}{l} \begin{array}{lll} {\delta \rho }^{i} & = & \rho^{i}(t)-\rho^{i}(t-1)\\ & = & \text{dot}({\vec{R}}^{i}(t)-{\vec{R}}_{r}(t),\vec{u}(t))-\text{dot}({\vec{R}}^{i}(t-1)-{\vec{R}}_{r}(t-1),\vec{u}(t-1))\\ & = & \text{dot}({\vec{R}}^{i}(t),\vec{u}(t))-\text{dot}({\vec{R}}^{i}(t-1),\vec{u}(t-1))-\text{dot}(\delta {\vec{R}}_{r}(t),\vec{u}(t))-\text{dot}({\vec{R}}_{r}(t-1),\delta \vec{u}(t)) \end{array}\\ i=1,...,k \end{array}$$

where dot(,) defines the vector dot product; R⃗*^i^* and R⃗*_r_* denote the position vectors of the satellite and the receiver in the *e* frame; $\vec{u}=\frac{{\vec{R}}^{i}-{\vec{R}}_{r}}{\left\|{\vec{R}}^{i}-{\vec{R}}_{r}\right\|}$ is the unit vector along the signal reception path.

According to the commutative and distributive laws of the vector dot product, Equation (16) yields the baseline of position change *δ*R⃗*_r_* as well as the change of the unit vector *δ*u⃗. With the satellite broadcast ephemeris, the satellite position vector R⃗*^i^* (*t* − 1) and R⃗*^i^* (*t*) can be easily estimated. In addition, the user’s previous position vector R⃗*_r_* (*t* − 1) has been optimally estimated by the filter of the integrated system, and the present position vector R⃗*_r_* (*t*) in Equation (16) can be initially determined by inertial navigation given a short time interval. The R⃗*_r_* (*t*) at this stage is not for the ultimate position solution at the present time *t* but for the upcoming *δ*R*_r_* estimation. Note that after the dot product operation, the desired unknown becomes the scalar *δ*R*_r_* instead of *δ*R⃗*_r_*.

Combining the Equations (15) and (16) with rearranged known/unknown quantities, the revised differential equation is expressed as:

(17) $$\delta {P^{\prime }}^{i}=-\text{cos}(\theta_{r}^{i})\cdot \delta R_{r}+c\cdot {\delta t}_{r}+{\delta \varepsilon }^{i}\;\;\;\;i=1,...,k$$

where:

*δP*′*^i^* is the pseudorange differential term compensated by the known quantities of the dot product operation;
$\theta_{r}^{i}$ represents the included angle between the vectors of *δ*R⃗*_r_* and u⃗.

With two or more available pseudorange observations, the unknown terms of *δ*R*_r_* and *δt_r_* can be derived by implementing the least-square fit. It is worth noting that the LOS measurements play an important role in accurate *δ*R*_r_* estimation as a contaminated measurement does not purely satisfy the above-mentioned satellite/user relationship and will give rise to a biased estimate of *δ*R*_r_*. In an effort to conduct multipath detection and mitigation in challenging GNSS environments, previous researchers have applied several approaches [32,33] by leveraging pseudorange, carrier phase or signal-to-noise ratio (SNR) measurements based on the test statistics. However, difficulties arise to road applications as a result of undesirable detection performance and required hardware upgrades [34]. Another easier option uses a weighting scheme by evaluating the satellite elevation angle and carrier-to-noise ratio (C/No) to restrain the multipath effect. With the synchronized GNSS and camera observations, the camera scale ambiguity can be finally resolved by the estimation of the baseline magnitude of position change *δ*R*_r_* between two GNSS epochs.

## 6. EKF Modeling and Implementation

To optimally implement sensor integration, several forms of non-linear filtering techniques were previously developed and validated, such as the EKF, unscented Kalman filter (UKF) as well as particle filter (PF). An EKF design linearizes the system and measurement models by considering a first-order Taylor series expansion at the predicted state estimate. This first-order approximation enables the linearized models to implement standard Kalman filter processing. In a typical UKF design, particularly for high non-linear instances, the selected sigma points through the unscented transform estimate the mean and covariance of the state vector. Based on the Bayesian estimation theory, the PF design is supposed to achieve a better solution compared to the EKF and UKF implementations if it is properly established. Nevertheless, the intense computational burden of the UKF and PF processing, one of their prominent shortcomings, brings about severe time-lag estimation and fatally hinders real-time road applications. On the other hand, for low-dynamic applications such as consumer-grade ground vehicle navigation, a time-efficient EKF-based sensor integration scheme requires limited computation and can agree well with the actual situation in terms of the accuracy in statistics [35,36]. The EKF approach is thus adopted as the proposed filter design.

As described previously, Equations (4) to (9) serve as the EKF discretized continuous-time model which is summarized as:

(18) $$\frac{dx(t)}{\mathrm{dt}}=\text{Fx}(t)+\text{Gw}(t),\;w(t)∼N(0,Q(t))$$

In Equation (18), F and G are the linearized dynamic matrix and input coefficient matrix with constant dimensionalities. Additive white Gaussian noise of the system is denoted as w(*t*) with a power spectral density Q(*t*) and given by:

(19) $$w(t)={\left[w_{f\;3\times 1}^{T}\;\;w_{\omega \;3\times 1}^{T}\;\;\mu_{f\;3\times 1}^{T}\;\;\mu_{\omega \;3\times 1}^{T}\right]}^{T}$$

where the four triads denote uncorrelated and correlated noises of the sensor biases. The 15-element system state vector is given by:

(20) $$x(t)={\left[{\delta P}_{3\times 1}^{n}{}^{T}\;\;{\delta V}_{3\times 1}^{n}{}^{T}\;\;{\delta \Psi }_{3\times 1}^{n}{}^{T}\;\;{\delta f}_{3\times 1}^{b}{}^{T}\;\;{\delta \omega }_{3\times 1}^{b}{}^{T}\right]}^{T}$$

where the triad perturbation terms of position, velocity, orientation and sensor biases are included.

As camera-derived navigation parameters, the rotation matrix *R_c_*(*t*) and translation unit vector *l⃗_c_*(*t*) require additional transformations to obtain the camera-based position and orientation under the *e* and *n* frames, correspondingly. Let $C_{b}^{n}(0)$ be the initial IMU orientation matrix and $C_{c}^{b}$ be the constant rotation matrix between the *c* and *b* frames. Assuming the origins of the *c* and *b* frames coincide, the camera-based orientation ${\tilde{C}}_{b}^{n}(t)$ can be expressed as:

(21) $${\tilde{C}}_{b}^{n}(t)=C_{b}^{n}(0)\cdot C_{c}^{b}\cdot {\left(V(t)\right)}^{T}\cdot {\left(C_{c}^{b}\right)}^{T}$$

where *V*(*t*) is the accumulated 3 × 3 camera rotation from the first image and satisfies:

(22) $$V(t)=R_{c}(t)\cdot V(t-1),\;V(1)=R_{c}(1)$$

Assuming ${\tilde{C}}_{b}^{n}(t)$ contains only WGN, the orientation error term *δ*Ψ*^n^* in Equation (20) correlates ${\tilde{C}}_{b}^{n}(t)$ as well as IMU-based orientation $C_{b}^{n}$ with the relationship of:

(23) $${\tilde{C}}_{b}^{n}(t)=[I_{3\times 3}+(\delta \Psi^{n}(t)\times )]\cdot C_{b}^{n}(t)+\Gamma_{3\times 3}(t)$$

where matrix Γ_3×3_ consists of the measurement noises. Rearranging Equation (23) yields:

(24) $${\delta \Psi }^{n}(t)\times =({\tilde{C}}_{b}^{n}(t)-C_{b}^{n}(t))\cdot {\left(C_{b}^{n}(t)\right)}^{-1}+\Gamma_{3\times 3}(t)$$

Let *a*_3,2_, *a*_1,3_ and *a*_2,1_ be the corresponding elements from the matrix of right-hand side multiplication and construct:

(25) $$A={\left[a_{3,2}\;a_{1,3}\;a_{2,1}\right]}^{T}$$

(26) $$\eta ={\left[\Gamma_{3,2}\;\Gamma_{1,3}\;\Gamma_{2,1}\right]}^{T}$$

According to Equations (24) to (26), the linear observation equation with respect to the orientation is finally given by:

(27) $$A(t)={\delta \Psi }^{n}(t)+\eta (t)$$

On the other hand, a camera-derived translation unit vector *l⃗_c_*(*t*) is transformed as:

(28) $${\vec{l}}_{n}(t)={\hat{C}}_{b}^{n}(t-1)\cdot C_{c}^{b}\cdot {\vec{l}}_{c}(t)$$

which means a unit vector expressed in the *n* frame. Note that the orientation matrix ${\hat{C}}_{b}^{n}$ does not refer to the present moment but the instant of previous image update and the caret ^ indicates the EKF-based estimation. By obtaining the translation magnitude *δ*R*_r_* which has been resolved in Section 5 by using differential LOS GNSS pseudoranges over epochs, the camera-based position is expressed as:

(29) $${\tilde{P}}^{e}(t)={\tilde{P}}^{e}(t-1)+\delta R_{r}\;(t)\cdot l_{n}\;(t)$$

In the above equation, *P̃^e^*(*t*) depicts the camera-based position at the present moment, and *P̂^e^*(*t* − 1) indicates the previous EKF position update, both expressed in the *e* frame. By subtracting the IMU-based position *P^e^*(*t*) from *P̃^e^*(*t*), the observation equation with respect to the position is established by:

(30) $$P^{e}(t)-{\tilde{P}}^{e}(t)={\delta P}^{n}(t)+\zeta (t)$$

where *ζ*(*t*) denotes the measurement noise. Combining Equations (27) and (30) yields the EKF measurement model as below:

(31) $$Y(t)=\text{Hx}(t)+v(t),\;v(t)∼N(0,R_{\mathrm{cov}}(t))$$

In Equation (31), the measurement and noise vectors are respectively given by:

(32) $$Y(t)={\left[{\left(P^{e}(t)-{\tilde{P}}^{e}(t)\right)}^{T}\;A{\left(t\right)}^{T}\right]}^{T}$$

(33) $$v(t)={\left[\zeta {\left(t\right)}^{T}\;\eta {\left(t\right)}^{T}\right]}^{T}$$

v(*t*) is assumed as WGN vector with a measurement noise covariance R*_cov_*(*t*). The measurement model matrix H is given by:

(34) $$H=[\begin{array}{ccccc} I_{3\times 3} & 0_{3\times 3} & 0_{3\times 3} & 0_{3\times 3} & 0_{3\times 3}\\ 0_{3\times 3} & 0_{3\times 3} & I_{3\times 3} & 0_{3\times 3} & 0_{3\times 3} \end{array}]$$

Following the standard EKF procedure, the state vector as well as its covariance matrix can be estimated and applied to ground vehicle navigation. After each update cycle, the state vector is reset to zero and refreshed by the next update operation.

**Figure 5.**
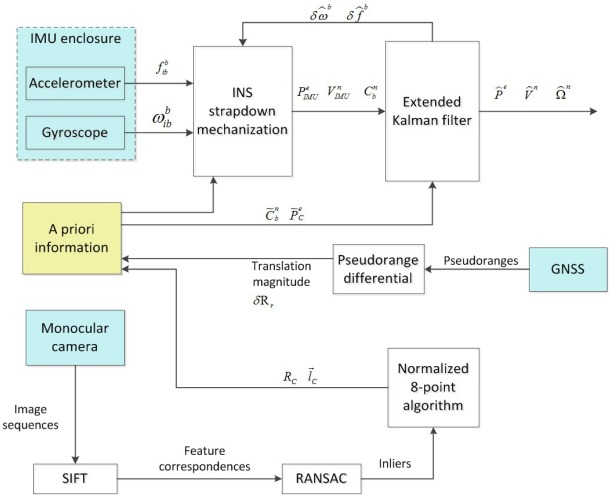
Flowchart of the proposed EKF-based architecture.

Figure 5 depicts an overall flowchart for integrating the camera, IMU and GNSS sensors through the EKF engine. The sensor units are colored cyan while white blocks indicate the utilized algorithms for intermediate parameter calculation. Between camera/GNSS data update, the vehicle motion is estimated by strapdown IMU mechanization. Once new camera/GNSS measurement data arrive, all derived navigation information is transformed in order to enable the EKF engine for accurate navigation solution and IMU sensor calibration. In such a method, the measurement model is straightforward to construct as relatively small/constant size of measurement elements are included in the EKF. The whole processing requires a relatively low computational load since covariance matrices have low dimensionality. Finally, a low-pass filter before implementing the EKF can be added to smooth the camera-derived rotation and translation parameters.

## 7. Experimental Description and Result Analysis

Our proposed integrated architecture is examined through a live test which was collected by a ground vehicle in an operational traffic environment in Málaga, Spain and is publicly available online [13]. The IMU and monocular forward-looking camera sensors were rigidly fixed on the vehicle rooftop. In order to provide a truth reference solution, three RTK GPS receivers were installed on the vehicle to generate centimeter-level positioning and orientation solutions. In total six drive segments have been accomplished, however the corresponding truth references were vulnerable to limited signal qualities. The *Campus_0L* segment had full range availability of truth reference and contains most common ground vehicle dynamics, such as acceleration, deceleration, stillness, forward driving, cornering, *etc.*, and it is therefore adopted in this paper to test our integrated design. Other sensors utilized during the drive test, such as laser scanners, are disregarded.

Table 1 presents some basic information of the overall drive test. In attempt to keep the recorded data from all sensors available during the whole test, we deliberately remove the beginning of the dataset when the vehicle was keeping static and the sensors were being powered on sequentially. The timespan in Table 1 is, consequently, approximately 20 s shorter than in the actual case.

Table 2 lists the individual IMU sensor specification of Xsens MTi enclosure [37]. Although this product enables 3D axes-based magnetometer to compensate for gyroscope drift, we ignore the magnetic field data and just make use of the 6-DOF raw angular rate and specific force measurements.

Table 3 summarizes the major intrinsic calibration parameters from the camera. There were actually two cameras of the same model mounted at both sides in front of the vehicle rooftop. Without loss of generality, only the left camera is employed in this paper and parameters in Table 3 are, accordingly, derived from the left one.

The parameters *f_x_* as well as *f_y_* denote the location of the principal point by means of pixel element on the image plane. The focal length is, similarly, defined in terms of pixel element in both x and y axes, and is hence depicted as *c_x_* and *c_y_*. The remainder of the parameters in Table 3 are radial and tangential distortions, respectively. In the *Campus_0L* segment, the rectified image frames are utilized instead of the originally captured ones to avoid the image projection offset and obtain accurate motion estimation parameters for sensor integration.

Although the *b* and *c* frames do not necessarily coincide with each other as in our case, the camera and IMU sensors are usually rigidly fixed to the vehicle and their relative translation and rotation parameters are, therefore, constant. The rotation calibration between the *b* and *c* frames can be accomplished according to Lobo [38] while the lever arm vector needs more effort to be accurately estimated. The difficulties of lever arm vector estimation arise due to the sensitivity of the selected target position and the geometric configuration [38]. Normally, in low dynamic situations with moderate maneuvers, particularly for ground vehicle applications, a mild lever arm has little impact little on the navigation solution and it does not necessarily require an accurate estimation [15,39]. Therefore, although both rotation and translation calibration results between the *b* and *c* frames are provided in the dataset, we simply disregard the lever arm effect caused by the translation and take only the relative rotation $C_{c}^{b}$ into consideration.

**Figure 6.**
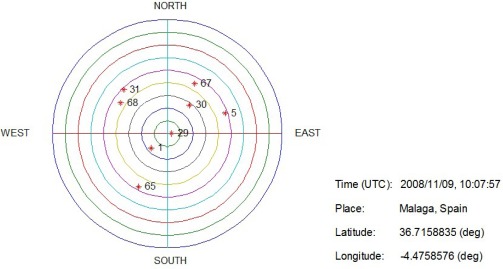
Sky plot of available GPS and GLONASS satellites with the elevation mask angle of 40 degree.

As described in Section 5, the scale factor ambiguity between every two successive image frames can be resolved using GNSS differential techniques, capable of obtaining the distance traveled over ground. In an effort to acquire more available GNSS measurements in potentially challenging environments, we take advantage of both GPS and GLONASS constellations for the pseudorange simulation. In typical challenging GNSS environments like urban downtown areas, low-elevation-angle signals are prone to severe multipath degradation or obstruction caused by nearby buildings. We, therefore, filter out those low-elevation-angle satellites from subsequent integration demonstrations. Figure 6 shows the sky plot of GPS/GLONASS satellites when the dataset was initially collected in Málaga with the elevation mask angle of 40 degree. The GPS pseudo random number (PRN) is between 1 and 32, while GLONASS orbital slot is designated within the range 51 to 74. Although the elevation mask angle is significantly higher than in most other applications, both constellations contributed 8 space vehicles for the integration processing. According to the differential operation described in Equation (15), most of the common error sources can be eliminated from the satellite-to-user geometry.

It is simulated in this paper that the LOS pseudorange measurements contain thermal noise with 0.25 m root-mean-square error (RMSE) during the whole *Campus_0L* segment. Note that the two constellations do not share the same coordinate system as well as time reference. In order to avoid newly introduced unknown quantities, the transformation between their coordinate frames has been implemented by using the approach presented by Cook [40] and, similarly, the system time offset can be extracted from the ephemerides of all modernized GLONASS-M satellites [41].

All the camera, IMU and GNSS measurements are timestamped. Instead of using the original 7.5 Hz frame rate, we deliberately downsample the visual measurements to 2.5 Hz to reduce the computational load and maintain preferable alignment with the 100 Hz IMU measurements for the EKF state update estimation. Since the IMU measurements were not necessarily synchronized with the image frames, an extrapolation process of IMU measurements is expected to establish synchronization consistency. According to You [42], a second-order polynomial extrapolation is utilized to balance the computational load and the accuracy of extrapolation. In order to demonstrate and assess how different sensory-integration schemes perform in challenging environments, we gradually reduce the number of visible GPS/GLONASS satellites with lower elevation angles over time and compare the performance between the tightly coupled GNSS/IMU integration and our proposed camera/IMU/GNSS scheme.

**Figure 7.**
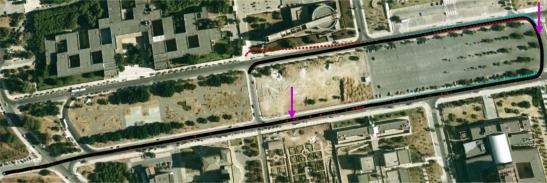
Google Earth visualization of the navigation trajectories (black line: truth trajectory, cyan line: camera/IMU/GNSS integration, red line: tightly coupled GNSS/IMU).

**Figure 8.**
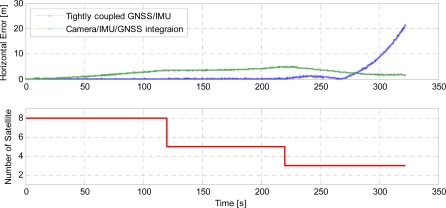
Comparison of horizontal error between the tightly coupled GNSS/IMU and camera/IMU/GNSS integrations in terms of reduced observability of GNSS measurements.

A Google Earth visualization of 1 Hz navigation trajectories of different integration schemes is qualitatively given in Figure 7 with common start/end times. The drive test started at the lower left and ended at the upper middle with the upside indicating the north direction. The black trajectory serves as the truth reference which was obtained by leveraging three RTK GPS receivers. The tightly coupled GNSS/IMU solution is illustrated in red and the vision/IMU/GNSS integration is shown as cyan line. Two purple arrows directly denote the place where the number of available satellites changes. A more quantitative comparison of the horizontal accuracy is shown in Figure 8 to reveal how the different integrated architectures react in terms of reduced observability of GNSS measurements in challenging environments.

Based on Figures 7 and 8, the tight coupling of GNSS and IMU measurements yields an accurate solution when an adequate number of satellites are available (above four). Meanwhile, the gradually accumulated error of proposed camera/IMU/GNSS approach reaches a maximum of 5 m before the number of visible satellites drops to three. The small systematic error mainly stems from erosions of unexpected pixel positioning error, camera calibration error, numerical instability, and remnants of the outliers after RANSAC processing and so forth. After the number of satellites drops to three, the tightly coupled GNSS/IMU integration still enables a continuous navigation solution with insufficient observability, however the estimation tends to sharply drift away from the truth reference over time and its horizontal error exceeds 20 m at the end of the test. Contrarily, compared to previous navigation results, the camera/IMU/GNSS integration yields a slightly better accuracy with only three utilizable satellites. The reasons for this phenomenon are twofold. First, based on Equation (17) which contains two unknown quantities, three observation equations still maintain a redundant constraint provided that the measurements are derived from LOS directions. More importantly, the systematic error of the image processing module gives rise to the reversal of the positioning error during the vehicle’s U-turn operation. As seen in Figure 7, the cyan line slightly tends to the left side of truth reference prior to the U-turn and therefore the same directionality of the systematic error makes the horizontal accuracy be partially compensated after the U-turn.

**Figure 9.**
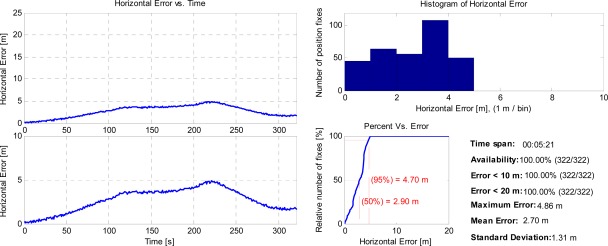
Horizontal positioning error statistics of the camera/IMU/GNSS integration.

According to the above-mentioned facts, the camera/IMU/GNSS integration brings us a potential advantage compared with the traditional tightly coupled GNSS/IMU technique in challenging environments with sparse GNSS observations. A more detailed horizontal positioning error analysis of the camera/IMU/GNSS integration is presented in Figure 9 with the same hypothesis.

The left side shows the horizontal positioning error in two different zoom levels. The right side shows the histograms, cumulative distribution curves and other related statistics. All the subfigures and corresponding statistics are based on 1 Hz solution. Refer to [1,43] which define these statistics/plots in detail.

**Figure 10.**
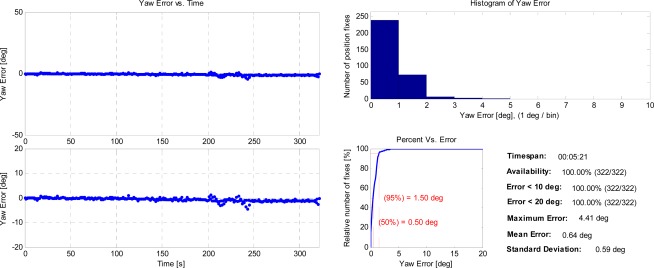
Yaw angle error statistics of the camera/IMU/GNSS integration.

Researchers sometimes refer to quaternions, rather than Euler angles, for orientation representation, particularly for high dynamic applications [14,15]. The quaternion expression avoids the gimbal lock phenomenon when the pitch angle approaches ±90 deg, and operates more efficiently compared with multiplications of direction cosine matrices. Whereas, since a ground vehicle is incapable of operating orthogonal to the ground plane, Euler angles do not suffer from the singularity problem and can provide an intuitive manner for the user to perceive the vehicle direction. We, therefore, choose roll, pitch and yaw angles for representing the vehicle’s orientation based on Tait-Bryan convention. Figure 10 shows the yaw angle error statistics of the camera/IMU/GNSS integration. Yaw jitters occur as a result of the abrupt change in the centrifugal force when the vehicle underwent the U-turn operation. Although the angle residual is maintained within 1 deg in a majority of the time during the test segment, the relatively low level of systematic error still can be observed from the zoomed-in subfigure due to the imperfection of the computer vision module. This further proves the reversal of the positioning error during the vehicle’s cornering as shown in Figure 8. It can be inferred that the accumulated position and orientation errors will gradually erode the navigation solution if the dataset is long enough without any other sources of corrections.

**Figure 11.**
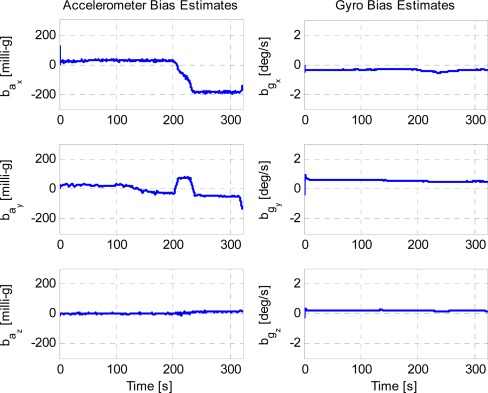
Accelerometer and gyroscope estimation based on the camera/IMU/GNSS integration.

In addition, the IMU sensor bias estimation is shown in Figure 11. Those large biases from both the accelerometer and gyroscope sensors inevitably cause erroneous IMU mechanization solutions in a matter of seconds without sensor calibration. The non-smoothly varying biases, particularly for the accelerometer components, gave rise to the unpredictability of the sensor errors. Simply averaging the sensor biases within even a few seconds may yield an inaccurate navigation solution, particularly when the vehicle dynamics significantly change with velocity and cornering stiffness. As seen in Figure 11, during the vehicle’s cornering, the accelerometer bias on x-axis dramatically dropped by approximately 200 milli-g. However, the actual sensor biases may be, to a certain extent, different from the estimation shown in Figure 11 in terms of the fidelities of the utilized filtering models [16].

**Figure 12.**
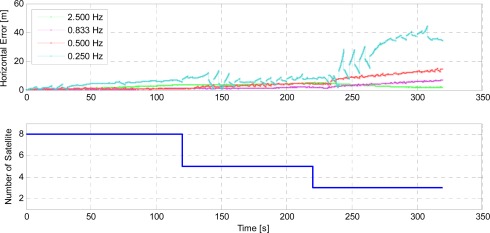
Horizontal positioning error comparison by using different image frame rate.

In an effort to balance the tradeoff between practical performance and computational cost, we deliberately reduce the image frame rate and examine the position solution of our integrated scheme by comparing with the truth reference. Figure 12 demonstrates a comparison of the horizontal positioning errors with different image frame rates based on the same GNSS observability as discussed previously.

As the interval of images is gradually enlarged, EKF filtering leans more on the inertial navigation alone which gives rise to larger location and orientation errors, and moreover, those fewer detected feature correspondences tend to degrade the reliability of camera motion estimation. The sawtooth-shaped curves therefore arise with worse accuracy. During the U-turn operation when the images partially overlap each other, SIFT/RANSAC algorithms acquire much fewer feature correspondences with lower frame rate, which results in abrupt change in positioning accuracy as seen in Figure 12, particularly for the low frame rates of 0.25 and 0.50 Hz. The worst situation may occur when no overlap can be found in two consecutive images. Consequently, the strategy to determine an image frame rate is primarily dependent on the vehicle dynamics, IMU quality and fidelity of utilized filtering models. In our case, 0.25 Hz frame rate reaches the lower limit as during the U-turn the gaps of the image overlap arise for a frame interval of approximately 4 s. It is worth noting that the vision-based error characteristics are relevant to the processed images and the systematic error therefore varies with the frame rate as seen in Figure 12, particularly for the time period after U-turn. A higher image frame rate tends to yield better integration accuracy at the cost of more excessive computational load, whereas the acceptable processing speed inevitably depends on the available computational capability. In *Campus_0L* segment, we used a 64-bit desktop computer with a 3 GHz processor to process the 2.5 Hz rate images with 1,024 × 768 resolution based on a MATLAB platform. Our implementation requires a few seconds of processing for each pair of images and tens of milliseconds for all other filtering routines and, consequently, has yet to achieve real-time functionality. Moore’s law, fortunately, has been appropriately indicating the rapid development of integrated circuits during the past decades and also has given us encouraging prediction on the exponentially growing processing capacity of semiconductor materials in the future. In addition, instead of using a CPU, a GPU module also facilitates speeding up the image processing task towards a real-time computation.

## 8. Conclusions

Aiming at taking advantage of the existing onboard technologies for ground vehicle navigation in challenging environments, this paper has developed an integrated camera/IMU/GNSS system based on the EKF design. The estimated motion parameters from successive image frames were utilized to compensate the drifting IMU sensor biases and maintain the accurate strapdown mechanization between the update of image data. For resolving the scale ambiguity problem of a monocular camera, GNSS differential technique provided an effective manner to extract the baseline magnitude of position change. Based on the EKF design, the proposed integrated system is able to provide 15-state high-bandwidth navigation solutions. Our implementation has been validated through a 5-minute long drive test which provided raw camera and IMU data as well as centimeter-accuracy truth reference. For a reasonable simulation of challenging GNSS environments, we gradually decreased the number of visible satellites with lower elevation angles over time. The experiment has provided results with high accuracies, a majority to the sub-degree level in the yaw angle and meter level in the horizontal plane. Furthermore, it was demonstrated that under the condition of sparse GNSS observations, the camera/IMU/GNSS integration potentially outperformed the tightly-coupled GNSS/IMU scheme which has yielded diverging navigation solutions. The camera/IMU/GNSS integration has also been tested with different image frame rates and the results revealed that the accuracy deteriorates with decreasing frame rates. Future work will further integrate GNSS measurements into the filtering architecture for better error growth control and automatically enable the loosely/tightly coupled GNSS/IMU integration in case relatively favorable GNSS conditions arise. Future work also includes a target objective of real-time functionality with revised computer vision algorithms on the GPU platform.

## Figures and Tables

**Table 1.** Basic information of the live drive test (Reproduced with permission from [13]).

***CAMPUS-0L* test details**	
Data-collection time (UTC)	2008/11/09, 10:07:57
Distance of travel	1,139 m
Timespan	322 s
IMU make/model	Xsens MTi
IMU output rate	100 Hz
Camera make/model	AVT Marlin F-131C
Image frame rate	7.5 Hz
Image resolution	1,024 × 768
Truth output rate	100 Hz

**Table 2.** Gyroscope and Accelerometer specification of Xsens MTi enclosure (Reproduced with permission from [37]).

	**Gyroscope**	**Accelerometer**
Drift rate	1 deg/s	0.02 m/s^2^
Noise	0.05 deg/s/√Hz	0.002 m/s^2^/√Hz
Bandwidth	40 Hz	30 Hz
Misalignment	0.1 deg	0.1 deg
Scale factor	—	0.03%

**Table 3.** Intrinsic camera parameters of the camera in use (Reproduced with permission from [13]).

**AVT Marlin F-131C camera**	
*f_x_* (pixel)	923.5295
*f_y_* (pixel)	922.2418
*c_x_* (pixel)	507.2222
*c_y_* (pixel)	383.5822
*k*1	−0.353754
*k*2	0.162014
*p*1	1.643379 × 10^−3^
*p*2	3.655471 × 10^−4^
